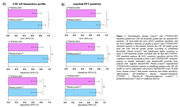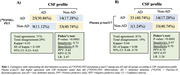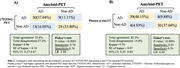# Plasma p‐tau217 outperforms [^18^F]FDG‐PET in identifying biological Alzheimer’s disease in atypical and early‐onset dementia

**DOI:** 10.1002/alz.093116

**Published:** 2025-01-09

**Authors:** Kely Monica Quispialaya Socualaya, Joseph Therriault, Antonio Aliaga, Arthur C. Macedo, Nesrine Rahmouni, Jaime Fernandez Arias, Jenna Stevenson, Yi‐Ting Wang, Cécile Tissot, Andrea L. Benedet, Nicholas J. Ashton, Thomas K Karikari, Tharick A. Pascoal, Jean‐Paul Soucy, Serge Gauthier, Paolo Vitali, Henrik Zetterberg, Pedro Rosa‐Neto

**Affiliations:** ^1^ Translational Neuroimaging Laboratory, The McGill University Research Centre for Studies in Aging, Montréal, QC Canada; ^2^ McGill University, Montreal, QC Canada; ^3^ Universidade Federal do Rio Grande do Sul, Porto Alegre, Rio Grande do Sul Brazil; ^4^ Lawrence Berkeley National Laboratory, Berkeley, CA USA; ^5^ Department of Psychiatry and Neurochemistry, Institute of Neuroscience and Physiology, The Sahlgrenska Academy, University of Gothenburg, Mölndal, Gothenburg Sweden; ^6^ University of Gothenburg, Gothenburg Sweden; ^7^ University of Gothenburg, Mölndal, Gothenburg Sweden; ^8^ University of Pittsburgh, Pittsburgh, PA USA; ^9^ Montreal Neurological Institute, McGill University, Montréal, QC Canada; ^10^ Translational Neuroimaging Laboratory, The McGill University Research Centre for Studies in Aging, Montreal, QC Canada; ^11^ McGill University, Montréal, QC Canada; ^12^ Translational Neuroimaging Laboratory, Montréal, QC Canada

## Abstract

**Background:**

Biomarkers promise to significantly improve the differential diagnosis of Alzheimer’s disease (AD). Plasma biomarkers, such as phosphorylated tau (p‐tau), have shown potential in diagnosing AD with high accuracy. Unlike the widely‐used [18 F]FDG‐PET diagnostic biomarker in clinical practice, plasma p‐tau is specific to AD and can provide an affordable and scalable diagnostic tool.

**Method:**

We conducted a retrospective analysis of individuals with atypical dementia, and/or early‐onset dementia cases, who were assessed at a specialized memory clinic. All participants underwent measurements of CSF Aβ42, p‐tau181, and total tau levels, as well as brain [^18^F]FDG‐PET scans and plasma p‐tau217 measurement. The [^18^F]FDG‐PET data was visually examined by two nuclear medicine experts to determine whether they were compatible with AD. CSF biomarker results were categorized as either AD biomarker positive or negative. Contingency analysis was performed to assess the relationships between PET scan interpretation and fluid biomarkers (plasma p‐tau217 and CSF p‐tau181 and Aβ42). CSF biomarker and amyloid‐PET were treated as the reference standard.

**Result:**

81 subjects with atypical dementia had CSF AD biomarker evaluation, [^18^F]FDG‐PET rating, and plasma p‐tau217 assessment. Both [^18^F]FDG‐PET and plasma p‐tau217 had high levels of agreement with reference standard AD biomarkers ([^18^F]FDG‐PET: 71%; plasma p‐tau217: 81%). Although both biomarkers had similar specificity for AD ([^18^F]FDG‐PET:70%, plasma p‐tau217:70%), plasma p‐tau217 had higher sensitivity for abnormal AD (97%). Overall accuracy was also higher for plasma p‐tau217 (AUC=84%, 95%CI = 0.75‐ 0.93). The same pattern of results was observed when using amyloid‐PET as the reference standard.

**Conclusion:**

Our study provides evidence that plasma p‐tau217 has excellent diagnostic performance for AD in individuals with early‐onset or atypical dementia evaluated in specialized settings. Nevertheless, the topographical information from [^18^F]FDG‐PET may give complementary information.